# ECG-GPT: considerations for advancing toward clinical implementation

**DOI:** 10.1093/ehjdh/ztag089

**Published:** 2026-06-16

**Authors:** Zekai Yu, Feiwei Qin

**Affiliations:** School of Computer Science and Technology, Hangzhou Dianzi University, Hangzhou, Zhejiang Province 310018, PR China; School of Computer Science and Technology, Hangzhou Dianzi University, Hangzhou, Zhejiang Province 310018, PR China

We read with great interest the article by Khunte *et al*.^1^ describing ECG-GPT, a vision encoder–decoder model that generates free-text diagnostic interpretations from 12-lead ECG images. The impressive scale of development and multinational validation, combined with the innovative image-based approach, represent a meaningful contribution to the field of AI-assisted electrocardiography. We offer the following considerations that may help guide future development and strengthen the clinical translation of this promising work.

The authors report diagnostic accuracy of 0.93–0.99 across 26 conditions, which is encouraging. However, given the low prevalence of several important conditions in the study cohorts, complementary metrics such as AUPRC and F1 score may offer a more complete picture of clinical utility.^2^ For conditions such as acute myocardial infarction (AUPRC 0.18) and right ventricular hypertrophy (AUPRC 0.10), these metrics suggest room for further optimization. Future reporting might benefit from foregrounding these class-imbalance-robust metrics alongside accuracy, particularly for rare but high-stakes diagnoses, so that readers can more readily assess the model's readiness for different clinical scenarios.

The authors thoughtfully acknowledge that cardiologist-confirmed diagnosis statements used as training labels ‘are not always completely accurate’. Building on this transparency, it may be valuable for future work to explore the extent to which the clinical workflow—where clinicians overread computer-generated pre-reads—introduces correlation between the training labels and existing algorithmic outputs. Additionally, a comparison with currently available computerized ECG interpretation systems would help clinicians understand the incremental value ECG-GPT offers and could further highlight the model's strengths.

One of ECG-GPT's most compelling features is its potential applicability in low-resource and community-based settings. To fully realize this promise, broader evaluation of the model's generative free-text capability across diverse populations and ECG acquisition systems would be informative. The current external validation on the CODE-15 dataset from Brazil is a valuable step, though it was limited to binary classification of six labels. Expanding this evaluation to include the quality and clinical coherence of free-text outputs in international settings would strengthen confidence in the model's generalizability—especially given that training data originated from a single US academic health system with a predominantly non-Hispanic White population (65.9%).

Finally, as the authors move toward clinical deployment, prospective evaluation of how ECG-GPT influences real-world decision-making—including time-to-diagnosis and downstream management—would complement the strong retrospective evidence presented and provide essential evidence of clinical impact.

We commend the authors for this important work and look forward to seeing ECG-GPT advance through these next stages of validation.

## Data Availability

Data sharing is not applicable to this article as no new data were created or analyzed in this study. **Compliance with ethical standards**
